# Omega-3 polyunsaturated fatty acids enrichment alters performance and immune response in infectious bursal disease challenged broilers

**DOI:** 10.1186/1476-511X-11-15

**Published:** 2012-01-25

**Authors:** Elham Maroufyan, Azhar Kasim, Mahdi Ebrahimi, Tech Chwen Loh, Mohd Hair Bejo, Hailemariam Zerihun, Fatemeh Hosseni, Yong Meng Goh, Abdoreza Soleimani Farjam

**Affiliations:** 1 Department of Animal Science, Universiti Putra Malaysia,43400 UPM Serdang, Selangor, Malaysia; 2 Department of Veterinary Preclinical Sciences, Universiti Putra Malaysia, 43400 UPM Serdang, Selangor, Malaysia; 3 Department of Veterinary Pathology and Microbiology, Universiti Putra Malaysia, 43400 UPM Serdang, Selangor, Malaysia; 4 Department of animal science, Faculty of Agriculture, Ferdowsi University of Mashhad, Mashhad, Iran

**Keywords:** *n-3 *PUFA, immune response, IBD, broiler

## Abstract

**Background:**

Infectious bursal disease (IBD) results in economic loss due to mortality, reduction in production efficiency and increasing the usage of antibiotics. This study was carried out to investigate the modulatory roles of dietary *n-3 *polyunsaturated fatty acids (PUFA) enrichment in immune response and performance of IBD challenged broiler chickens.

**Methods:**

A total of 300 day old male broiler chicks were assigned to four dietary *n-3 *PUFA ascending levels as the treatment groups (T1: 0.5; T2: 8.0; T3: 11.5; T4: 16.5) using combinations of tuna oil and sunflower oil. All diets were isocaloric and isonitrogenous. On day 28, all birds were challenged with IBD virus. Antibody titer, cytokine production, bursa lesion pre and post-challenge and lymphoid organ weight were recorded.

**Results:**

On d 42 the highest body weight was observed in the T2 and T3 and the lowest in T4 chickens. Feed conversion ratio of the T2 broilers was significantly better than the other groups. Although productive parameters were not responded to the dietary n-3 PUFA in a dose-dependent manner, spleen weight, IBD and Newcastle disease antibody titers and IL-2 and IFN-γ concentrations were constantly elevated by n-3 PUFA enrichment.

**Conclusions:**

Dietary n-3 PUFA enrichment may improve the immune response and IBD resistance, but the optimum performance does not coincide with the optimum immune response. It seems that dietary n-3 PUFA modulates the broiler chicken performance and immune response in a dose-dependent manner. Thus, a moderate level of dietary n-3 PUFA enrichment may help to put together the efficiency of performance and relative immune response enhancement in broiler chickens.

## Background

There has been fast growth in the global interest to the effects of diet on various aspects of immune function. Typically, different research groups have assessed different nutrients, each using different animal models. Most of these models focus on selected aspects of immune function without assessing the other functions. It is therefore always difficult to understand the broader modulating influence for instance on the productive parameters. In modern poultry industry, however, within commonly used feed there is an oversupply of *n-6 *polyunsaturated fatty acids (PUFA) and an undersupply of *n-3 *PUFA, causing an imbalance in the corn-soybean diet. Consumption of *n-3 *PUFA, resulted in incorporation of these fatty acids into lipid membrane of all tissues, including cells of the immune system [1,2]. Long chain *n-3 *PUFA showed to improve the immune response and reduce inflammation in different species such as chicken, mice and fish [3-5].

On the other hand, infectious bursal disease (IBD) is one of the most important chicken diseases that have emerged in the last 30 years. IBD viral infection suppresses the immune system in young chickens, making birds sensitive to other diseases [6-8]. In poultry production, it is very important to improve immunity for prevention of infectious diseases. Minimizing immunosuppression and its impact is an important factor for success in the broiler production. In vivo and in vitro studies have shown that the target cell in IBD is IgM-bearing B lymphocyte. The acute lytic phase of the virus is associated with a reduction in circulating IgM^+ ^cells [8-11]. Immunoglobulin M is the major class of antibody found in serum, in the primary immune response (first encounter of antigen). Supplementation of dietary n-3 PUFA has shown to increase IgM level, suggesting an immuno-stimulating property of n-3 PUFA. immunoglobulins production by β cells and interferon-γ (IFN-γ) is facilitated by interlukin-2 (IL-2). This elevation is more pronounced in docosahexaenoic acid (DHA) rich diet than eicosapentaenoic acid (EPA) rich diet [12]. Another study showed that IgG and IgE response increased to ovalbumin in rats fed a high fish oil diet [13]. It has also demonstrated that *n-3 *PUFA from fish oil have an antiviral effect in mice [14,15]. The *n-3 *PUFA in fish oil can affect immune cell activation in both the innate and specific immune systems in different species [4,16-18]. Therefore, diet enrichment with n-3 PUFA consider as an alternative to enhance immune response and disease resistance. Among common sources of fish oil used in biomedical research, tuna oil has an interesting balance of n-3 PUFA and it contains much more DHA than EPA [19,20].

As for the chicken nutrition and immune response, to date, there is no report on immunomodulatory effects of dietary n-3 PUFA under IBD challenge in broiler chickens. Therefore, this study aimed to investigate the influence of dietary n-3 PUFA enrichment on immune response and performance of IBD challenged broiler chickens.

## Results

### Nutrient Composition and Fatty Acids Analysis

Nutrient composition is shown in Table 1 and fatty acid profile of the experimental diets and oils are shown in Table 2 and 3. Linoleic acid (C18:2 *n-6*) was the main *n-6 *PUFA in the diets and it was decreased as the fish oil increased. An opposite trend was observed for EPA and DHA contents while α-linolenic acid (C18:3*n-3*) remained unchanged. The resulting DHA content of the diets were 0, 5.6, 8.17 and 11.93 for T1 to T4, respectively. Influence of treatment diets on plasma fatty acid composition on d 28 and 42 are shown in Table 4 and 5. It was clearly indicated that the incorporation of fish oil into diet significantly increased the *n-3 *PUFA in plasma. On d 28 and 42 the highest plasma *n-3 *PUFA was observed in the T4 birds diet while the highest plasma *n-6 *PUFA was measured in T2 birds. The same results were observed in breast muscle on d 42 (Table 6).

**Table 1:** Nutrient composition of experimental diets (%).

	1-21 days				22-42 days			
	
Ingredients	T1^†^	T2	T3	T4	T1	T2	T3	T4
Corn	44.91	45.61	45.75	45.86	49.90	51.45	51.49	51.54
Soybean meal	43.85	43.73	43.59	43.48	38.67	38.43	38.39	38.34
Palm oil	6.58	0.00	0.00	0.00	7.31	0.00	0.00	0.00
Sunflower oil	0.00	3.50	2.00	0.50	0.00	3.50	2.00	0.50
Tuna oil	0.00	2.50	4.00	5.50	0.00	2.50	4.00	5.50
Di calcium phosphate	1.91	1.91	1.91	1.91	1.77	1.77	1.77	1.77
Limestone	1.20	1.20	1.20	1.20	1.06	1.06	1.06	1.06
Salt	0.44	0.44	0.44	0.44	0.31	0.31	0.31	0.31
Vitamin premix	0.30	0.30	0.30	0.30	0.30	0.30	0.30	0.30
Mineral premix	0.30	0.30	0.30	0.30	0.30	0.30	0.30	0.30
DL-Methionine	0.25	0.25	0.25	0.25	0.23	0.23	0.23	0.23
Lysine	0.26	0.26	0.26	0.26	0.15	0.15	0.15	0.15
Calculated composition (%)								
Crude protein	22.00	22.00	22.00	22.00	20.50	20.50	20.50	20.50
ME (Kcal/kg)	3080	3080	3080	3080	3150	3150	3150	3150
Available phosphorus	0.45	0.45	0.45	0.45	0.42	0.42	0.42	0.42
Calcium	1.00	1.00	1.00	1.00	0.90	0.90	0.90	0.90
Methionine	0.55	0.55	0.55	0.55	0.50	0.50	0.50	0.50
Lysine	1.20	1.20	1.20	1.20	1.00	1.00	1.00	1.00
Na	0.20	0.20	0.20	0.20	0.15	0.15	0.15	0.15

^† ^Dietary n-3 PUFA level of 0.5 (T1), 8.0 (T2), 11.5 (T3), 16.5% (T4).

**Table 2:** Fatty acid composition of dietary treatments (percentage of total fatty acids).

	Treatments			
	
Fatty acids	T1^†^	T2	T3	T4
14:0	0.8	0.9	1.8	2.5
15:0	-	-	-	0.7
15:1	-	-	-	-
16:0	32.7	15.7	17.3	19.5
16:1	-	1.4	2.4	3.3
17:0	-	0.9	1.0	1.4
17:1	-	-	0.6	0.8
18:0	4.1	4.9	4.9	5.6
18:1	39.2	27.8	26.9	23.3
18:2 *n-6*	21.9	38.8	31.9	24.8
18:3 *n-3*	-	0.5	0.6	0.6
20:4 *n-6*	-	0.6	0.7	1.1
20:5 *n-3*	-	1.5	2.3	3.2
22:5 *n-3*	-	-	0.6	0.8
22:6 *n-3*	-	5.6	8.2	11.9
**SFA**	38.2	23.2	25.9	30.0
**UFA**	61.8	76.8	74.1	69.9
**MUFA**	39.5	29.5	29.9	27.5
**PUFA *n-3***	0.5	8.0	11.5	16.5
**PUFA *n-6***	21.9	39.4	32.6	25.9
**UFA: SFA**	1.6	3.5	2.9	2.3
**PUFA: SFA**	0.6	2.1	1.7	1.4
***n-6*: *n-3***	51.7	5.1	2.8	1.6

^† ^Dietary *n-3 *PUFA level of 0.5 (T1), 8.0 (T2), 11.5 (T3), 16.5% (T4).

**Table 3:** Fatty acid composition of dietary treatments (%).

Fatty acids	Palm oil	Tuna oil	Sunflower oil
14:0	1	3.1	nd
16:0	45	22.8	7
18:0	4	6.7	5
18:1 *n-9*	40	17.7	19
18:2 *n-6 *	10	1.6	68
18:3 *n-3*	-	2.3	1
20:5 *n-3*	-	4.6	-
22:6 *n-3*	-	18.3	-
PV^† ^(mEq/Kg)	0.9	1.4	1.1

^†^PV = Peroxide value. nd = not detected.

**Table 4:** Influence of dietary treatments on plasma fatty acid composition at 28 days of age in broiler chickens (%).

	Treatments			
	
Fatty acids	T1^†^	T2	T3	T4
14:0	0.46 ± 0.03^b^	0.49 ± 0.01^b^	0.67 ± 0.1^a^	0.84 ± 0.1^a^
15:0	1.17 ± 0.2	1.37 ± 0.1	1.01 ± 0.2	1.23 ± 0.2
16:0	21.41 ± 0.5^b^	20.84 ± 0.8^b^	22.37 ± 0.6^ab^	23.99 ± 0.4^a^
16:1	1.66 ± 0.7	0.61 ± 0.1	1.02 ± 0.1	1.14 ± 0.1
17:0	0.82 ± 0.2	0.95 ± 0.2	0.97 ± 0.2	0.84 ± 0.1
18:0	12.78 ± 0.2	14.09 ± 0.5	12.71 ± 0.3	13.10 ± 0.6
18:1 *n-9*	29.15 ± 1.5^a^	15.36 ± 1.3^b^	15.53 ± 0.7^b^	14.67 ± 0.9^b^
18:2 *n-6*	24.70 ± 0.7^b^	30.06 ± 0.9^a^	26.41 ± 1.3^a^	21.19 ± 1.2^c^
18:3 *n-3*	0.64 ± 0.04	0.48 ± 0.03	0.65 ± 0.1	0.69 ± 0.1
20:4 *n-6*	4.12 ± 0.9^ab^	4.61 ± 0.5^a^	3.57 ± 0.3^ab^	2.95 ± 0.3^b^
20:5 *n-3*	-	1.71 ± 0.1^c^	3.21 ± 0.2^b^	3.95 ± 0.2^a^
22:6 *n-3*	-	6.92 ± 0.1^c^	9.33 ± 0.4^b^	12.52 ± 0.6^a^
**SFA**	38.68 ± 0.7^b^	39.26 ± 0.5^ab^	39.10 ± 0.7^b^	41.69 ± 0.5^a^
**UFA**	61.32 ± 0.7^a^	60.74 ± 0.5^a^	60.90 ± 0.7^a^	58.31 ± 0.5^b^
**MUFA**	31.86 ± 2.2^a^	16.95 ± 1.3^b^	17.72 ± 0.8^b^	17.01 ± 1.0^b^
**PUFA *n-3***	0.64 ± 0.04^d^	9.12 ± 0.2^c^	13.20 ± 0.5^b^	17.16 ± 0.8^a^
**PUFA *n-6***	28.82 ± 1.6^b^	34.67 ± 1.4^a^	29.98 ± 1.2^b^	24.14 ± 1.3^b^
**UFA: SFA**	1.59 ± 0.1	1.55 ± 0.03	1.56 ± 0.05	1.40 ± 0.03
**PUFA: SFA**	0.76 ± 0.03^b^	1.12 ± 0.1^a^	1.11 ± 0.04^a^	0.99 ± 0.04^a^
***n-6*: *n-3***	45.26 ± 1.9^a^	3.80 ± 0.1^b^	2.29 ± 0.2^c^	1.43 ± 0.1^d^

^† ^Dietary *n-3 *PUFA level of 0.5 (T1), 8.0 (T2), 11.5 (T3), 16.5% (T4). n = 8 birds/treatment.

^a-d ^Means with different superscripts within a row differ significantly (P < 0.05).

**Table 5:** Influence of dietary treatments on plasma fatty acid composition at 42 days of age in broiler chickens (%).

	Treatments			
	
Fatty acids	T1^†^	T2	T3	T4
14:0	0.48 ± 0.1^b^	0.61 ± 0.04^ab^	0.77 ± 0.1^a^	0.89 ± 0.1^a^
15:0	0.83 ± 0.1^b^	0.62 ± 0.2^b^	0.84 ± 0.2^b^	1.50 ± 0.1^a^
16:0	21.71 ± 0.7^b^	23.80 ± 0.4^ab^	24.94 ± 1.0^a^	25.45 ± 0.4^a^
16:1	3.24 ± 0.5^a^	1.54 ± 0.4^b^	1.30 ± 0.3^b^	1.47 ± 0.1^b^
17:0	0.56 ± 0.1	1.02 ± 0.2	1.04 ± 0.3	1.10 ± 0.2
18:0	15.21 ± 0.7^a^	13.87 ± 0.4^ab^	12.45 ± 0.6^b^	12.31 ± 0.6^b^
18:1 *n-9*	32.28 ± 1.2^a^	19.51 ± 1.1^ab^	15.54 ± 0.5^c^	17.90 ± 1.0^b^
18:2 *n-6*	19.24 ± 1.1^bc^	23.41 ± 1.8^a^	21.66 ± 1.3^ab^	15.92 ± 1.0^c^
18:3 *n-3*	0.42 ± 0.1b^c^	0.48 ± 0.03^bc^	0.58 ± 0.1^ab^	0.66 ± 0.1^a^
20:4 *n-6*	3.81 ± 0.2^a^	2.93 ± 0.6^ab^	2.72 ± 0.5a^b^	1.94 ± 0.1^b^
20:5 *n-3*	-	2.32 ± 0.1^c^	3.75 ± 0.3^b^	5.18 ± 0.7^a^
22:6 *n-3*	-	7.63 ± 0.9^b^	11.07 ± 0.7^a^	13.03 ± 0.6^a^
**SFA**	40.06 ± 1.0	41.03 ± 0.8	41.99 ± 1.1	42.66 ± 0.3
**UFA**	59.94 ± 1.0	58.97 ± 0.8	58.01 ± 1.1	57.34 ± 0.3
**MUFA**	36.47 ± 1.7^a^	22.21 ± 1.b^b^	18.23 ± 0.5^b^	20.61 ± 1.0^b^
**PUFA *n-3***	0.42 ± 0.1^d^	10.43 ± 0.9^c^	15.39 ± 0.8^b^	18.87 ± 0.7^a^
**PUFA *n-6***	23.05 ± 1.2^a^	26.33 ± 1.5^a^	24.39 ± 1.5^a^	17.86 ± 1.0^b^
**UFA: SFA**	1.51 ± 0.1	1.44 ± 0.1	1.39 ± 0.1	1.34 ± 0.0
**PUFA: SFA**	0.59 ± 0.03^b^	0.90 ± 0.07^a^	0.96 ± 0.1^a^	0.86 ± 0.03^a^
***n-6*: *n-3***	62.29 ± 11.1^a^	2.58 ± 0.2^b^	1.61 ± 0.2^c^	0.95 ± 0.1^c^

^† ^Dietary *n-3 *PUFA level of 0.5 (T1), 8.0 (T2), 11.5 (T3), 16.5% (T4). n = 8 birds/treatment.

^a-d ^Means with different superscripts within a row differ significantly (P < 0.05).

**Table 6:** Influence of dietary treatments on breast muscle fatty acid composition at 42 days of age in broiler chickens (%).

	Treatments			
	
Fatty acids	T1^†^	T2	T3	T4
14:0	0.66 ± 0.04^c^	0.79 ± 0.03^bc^	0.90 ± 0.05^ab^	1.07 ± 0.06^a^
15:0	0.45 ± 0.08	0.42 ± 0.04	0.53 ± 0.09	0.56 ± 0.06
16:0	23.50 ± 0.51	21.82 ± 0.46	22.03 ± 0.35	21.64 ± 1.48
16:1	2.81 ± 0.45^a^	1.29 ± 0.14^c^	1.63 ± 0.26^bc^	2.51 ± 0.28^ab^
17:0	0.37 ± 0.03	0.60 ± 0.10	0.66 ± 0.07	0.71 ± 0.10
18:0	10.01 ± 0.51	11.81 ± 0.36	11.71 ± 0.68	11.92 ± 0.89
18:1 *n-9*	35.11 ± 1.00^a^	21.26 ± 0.78^b^	20.96 ± 1.39^b^	19.53 ± 1.57^b^
18:2 *n-6 *	16.47 ± 0.41^b^	20.57 ± 0.90^a^	16.24 ± 1.06^b^	14.77 ± 1.49^b^
18:3 *n-3 *	0.45 ± 0.03	0.42 ± 0.03	0.42 ± 0.08	0.59 ± 0.08
20:4 *n-6 *	5.67 ± 0.70^ab^	6.17 ± 0.41^a^	5.11 ± 0.77^ab^	3.95 ± 0.61^b^
20:5 *n-3 *	-	1.20 ± 0.16^b^	2.24 ± 0.37^a^	2.83 ± 0.14^a^
22:6 *n-3 *	-	8.26 ± 0.74^b^	13.13 ± 0.82^a^	14.84 ± 1.18^a^
**SFA**	35.99 ± 0.52	37.04 ± 0.90	37.27 ± 1.01	37.32 ± 1.10
**UFA**	64.01 ± 0.52	62.96 ± 0.90	62.73 ± 1.01	62.68 ± 1.10
**MUFA**	39.80 ± 1.23^a^	24.91 ± 0.73^b^	24.66 ± 1.51^b^	24.04 ± 1.39^b^
**PUFA *n-3***	0.45 ± 0.03^c^	9.88 ± 0.82^b^	15.79 ± 0.96^a^	18.26 ± 1.19^a^
**PUFA *n-6***	23.76 ± 0.96^b^	28.17 ± 0.81^a^	22.28 ± 0.86^b^	20.38 ± 1.67^b^
**UFA: SFA**	1.78 ± 0.04	1.71 ± 0.07	1.69 ± 0.08	1.69 ± 0.08
**PUFA: SFA**	0.67 ± 0.02^b^	1.03 ± 0.05^a^	1.02 ± 0.03^a^	1.05 ± 0.09^a^
***n-6*: *n-3***	54.29 ± 4.79^a^	2.96 ± 0.29^b^	1.45 ± 0.15^c^	1.14 ± 0.12^c^

^† ^Dietary *n-3 *PUFA level of 0.5 (T1), 8.0 (T2), 11.5 (T3), 16.5% (T4). n = 8 birds/treatment.

^a-d ^Means with different superscripts within a row differ significantly (P < 0.05).

### Performance Parameters

There were no effects of dietary n-3 PUFA enrichment on Body weight, feed intake and FCR on d 21 (Table 7). However, on d 42, birds in T4 group that received the highest n-3 PUFA showed the lowest body weight (P < 0.05). A superior FCR was recorded for the birds of group T2. Feed intake was not affected by the dietary n-3 PUFA enrichment throughout the study (P > 0.05).

**Table 7:** Effect of *n-3 *PUFA enrichment on performance and mortality of IBD challenged broiler chickens.

	1-21 days			1-42 days			
		
Treatments	Body weight (g)	Feed intake (g/bird)	FCR	Body weight (g)	Feed intake (g/bird)	FCR	Mortality (%)
**T1^†^**	798 ± 10	1397 ± 25	1.75 ± 0.02	2215 ± 30^ab^	4740 ± 87	2.05 ± 0.03^a^	7 ± 2
**T2**	788 ± 11	1366 ± 33	1.75 ± 0.03	2276 ± 28^a^	4568 ± 74	1.94 ± 0.03^b^	7 ± 2
**T3**	801 ± 10	1335 ± 9	1.72 ± 0.03	2251 ± 28^a^	4689 ± 66	1.97 ± 0.03^ab^	7 ± 2
**T4**	783 ± 9	1403 ± 16	1.78 ± 0.03	2147 ± 29^b^	4570 ± 40	2.05 ± 0.03^a^	8 ± 2

^† ^Dietary *n-3 *PUFA level of 0.5 (T1), 8.0 (T2), 11.5 (T3), 16.5% (T4). n = 75 birds/treatment.

^a-b ^Means within a column with different letters differ significantly (P < 0.05).

### Lymphoid Organ Weight

Bursa of Fabricius weight was not influenced by n-3 PUFA enrichment (P > 0.05) (Table 8). Spleen weight 7 d after challenge was increased steadily by dietary treatment from T1 to T4 where the lowest was recorded in T1 and the highest in T4. There were no significant differences in spleen weight pre-challenge or 14 days after challenge.

**Table 8:** Effect of *n-3 *PUFA enrichment on relative lymphoid organ weight in IBD challenged broiler chickens.

	Spleen			Bursa of Fabricius		
	
Treatment	Pre-challenge	7 days post-challenge	14 days post-challenge	Pre-challenge	7 days post-challenge	14 days post-challenge
**T1^†^**	**0.10 ± 0.004**	**0.06 ± 0.003^b^**	**0.08 ± 0.008**	**0.18 ± 0.005**	**0.11 ± 0.009**	**0.04 ± 0.004**
**T2**	**0.10 ± 0.010**	**0.09 ± 0.009^ab^**	**0.08 ± 0.010**	**0.23 ± 0.007**	**0.10 ± 0.010**	**0.05 ± 0.005**
**T3**	**0.11 ± 0.008**	**0.09 ± 0.008^ab^**	**0.09 ± 0.011**	**0.22 ± 0.02**	**0.11 ± 0.010**	**0.05 ± 0.002**
**T4**	**0.09 ± 0.009**	**0.12 ± 0.008^a^**	**0.09 ± 0.010**	**0.22 ± 0.02**	**0.10 ± 0.009**	**0.04 ± 0.004**

^† ^Dietary *n-3 *PUFA level of 0.5 (T1), 8.0 (T2), 11.5 (T3), 16.5% (T4). n = 8 birds/treatment.

^a-b ^Means within a column with different letters differ significantly (P < 0.05).

### Histopathological Lesion Score

The Effect of dietary *n-3 *PUFA enrichment on lesion score of bursa in IBD challenged broiler chickens is presented in Table 9. Dietary treatment was not affected the bursa of Fabricius tissue pathologically throughout the study as measured by lesion scoring (P > 0.05).

**Table 9:** Effect of *n-3 *PUFA enrichment on histopathological changes (lesion score) of bursa in IBD challenged broiler chickens.

Treatment	Pre-challenge	7 days post-challenge	14 days post-challenge
**T1^†^**	0.5 ± 0.2	2.0 ± 0.4	3.8 ± 0.2
**T2**	0.3 ± 0.2	3.2 ± 0.5	3.0 ± 0.7
**T3**	0.3 ± 0.2	3.0 ± 0.3	3.7 ± 0.2
**T4**	0.6 ± 0.2	2.2 ± 0.3	3.5 ± 0.3

^† ^Dietary *n-3 *PUFA level of 0.5 (T1), 8.0 (T2), 11.5 (T3), 16.5% (T4).

n = 8 birds/treatment.

### Serology

**Figure 1:**
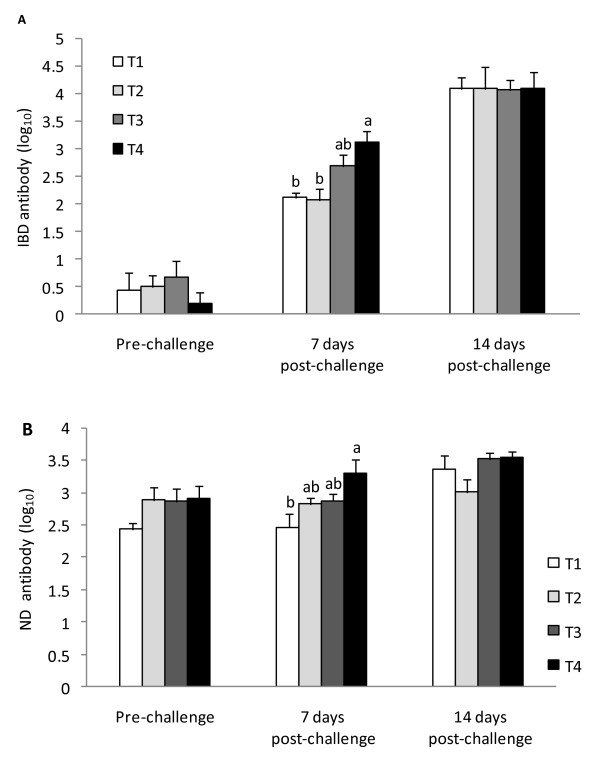
**Effects of *n-3 *PUFA enrichment on serum IBD (A) and ND (B) antibody titers (log_10_) in IBD challenged broiler chickens**. Dietary *n-3 *PUFA levels were as follows: 0.5 (T1), 8.0 (T2), 11.5 (T3), 16.5% (T4). Measurements were on 28 days of age (Pre-challenge) and 7 and 14 days after the IBD challenge. ^a-b ^Means with no common letter within sampling time subgroup differ *P *< 0.05. n = 10 birds/treatment.

The effect of dietary *n-3 *PUFA enrichment on serum antibody titer (log_10_) in broilers vaccinated against ND and challenged with IBD virus is shown in Figure 1. Serum IBD and ND antibody titers were not influenced by treatments pre-challenge on d 28 and 14 days after challenge. However, there was a significant alteration in both antibody titers seven days after IBD challenge. Interestingly, this alteration was along with the ascending trend of n-3 PUFA enrichment. The lowest and highest IBD and ND antibody titers were in T1 and T4 chickens, respectively.

### Cytokine Production

The IL-2 and IFN-γ levels increased along with the *n-3 *PUFA enrichment pre and 2 days post-challenge from T2 group to T4 (Table 10). However, there was no difference in IFN-γ level 2 days post-challenge and IL-2 and IFN-γ 7 days post-challenge. Surprisingly, both IL-2 and IFN-γ were significantly higher in T1 than T2 before challenge.

**Table 10:** Effects of *n-3 *PUFA enrichment on serum IL-2 and INF-γ concentrations in IBD challenged broiler chickens.

	IL-2 (ng/ml)			INF-γ (ng/ml)		
	
Treatment	Pre-challenge	2 days post-challenge	7 days post-challenge	Pre-challenge	2 days post-challenge	7 days post-challenge
**T1^†^**	7.9 ± 1.0^a^	7.2 ± 0.7^b^	5.8 ± 1.7	5.7 ± 1.0^a^	7.9 ± 1.3	5.3 ± 1.9
**T2**	2.9 ± 0.7^b^	5.1 ± 1.5^b^	6.8 ± 1.0	2.1 ± 0.7^b^	6.9 ± 0.9	5.7 ± 1.1
**T3**	6.5 ± 1.2^a^	6.3 ± 0.6^b^	4.5 ± 1.3	3.6 ± 0.7^ab^	7.4 ± 2.0	4.4 ± 1.6
**T4**	8.4 ± 0.9^a^	10.6 ± 1.0^a^	5.3 ± 0.6	6.2 ± 0.9^a^	7.7 ± 1.9	5.8 ± 1.9

^† ^Dietary *n-3 *PUFA level of 0.5 (T1), 8.0 (T2), 11.5 (T3), 16.5% (T4). n = 5 birds/treatment.

^a-b ^Means within a column with different letters differ significantly (P < 0.05).

## Discussion

Fats and oils are usually provided for chicken as dietary energy source to enhance the productivity and fulfill the high energy demand of fast growing broilers. Among the different fats and oils, the high *n-6 *PUFA sources are cheaper and easier available. Incorporation of these sources resulted in high concentration of the *n-6 *PUFA in the meat or egg product, and less EPA, (20:5 *n-3*), docosapentaenioc (DPA 22:5 *n-3*) and DHA (22:6 *n-3*) [21]. On the other hand, dietary n-3 PUFA enrichment and lowering *n-6*: *n-3 *PUFA ratio alters the fatty acid profile of meat toward long chain PUFA [22,23]. In agreement, current study results showed that the total *n-3 *PUFA, EPA and DHA of plasma and breast muscle significantly increased along with ascending n-3 PUFA inclusion from T1 to T4. There was a relatively high level of arachidonic acid (AA, 20:4 *n-6*) to linoleic acid (LA, 18:2 *n-6*) in plasma of T1 on day 28 and 42. This phenomenon can be explained by the absence of linolenic acid (LNA, 18:3 *n-3*) in the diet of this group, while the other groups have LNA of 0.5, 0.6 and 0.6% (Table 2). Simopoulos [16] indicated that there is a competition between LA and LNA for conversion to the long-chain PUFA because they share the same enzymes and LNA is the preferred substrate. Moreover, there was no detectable DHA in plasma of T1 as their representative diet was also free of EPA and DHA and their precursor LNA.

Dietary n-3 PUFA enrichment also increased body weight and improved FCR as measured in T2 and T3 at 42 days of age. This may be due to the superior digestibility of unsaturated fatty acid compared to saturated type [24-27]. Newman [21] showed the modulatory effects of dietary *n-3 *and *n-6 *PUFA FCR in avian metabolism through the modulation of lipid deposition and oxidation. However, the enhancement was not observed in highest n-3 PUFA (T4) group, leading to the postulation that effect of n-3 PUFA enrichment is a dose-dependent response. Hulan [28] reported that isoenergetic and isonitrogenous diets enriched with redfish meal and redfish oil led to lower body weights than the control diet. They attributed this result to lower palatability and higher calcium levels, although no palatability problem as a result of n-3 PUFA supplementation in the diet was found in the present study. Evidently, significant differences due to dietary treatment are absent from the feed intake throughout the experimental period. In any case, in accordance Korver [29] demonstrated that low levels of dietary fish oil were more efficient than high levels in improving broiler growth rate and FCR, even when the birds were undergoing an inflammatory response.

Immune tissue development can in some cases reflect immune system response and functionality. In the current study, there was no affect of dietary treatment on the bursa of Fabricius weight and lesion score, but spleen weight increased as the level of n-3 PUFA in diet increased 7 days post-challenge. Some other studies reported that feeding PUFA to chickens [30] and mice [31] results in increased spleen weights. In the study of Wang [30], the author used single-comb White Leghorn layers fed sunflower oil, animal oil, linseed oil, or fish oil at 5% (wt/wt). The results demonstrated that chicks fed the 3 PUFA-rich diets had significantly higher weights of the thymus, spleen, and bursa compared with those of chicks fed the diet with animal oil. In our study, the spleen enlargement due to dietary n-3 PUFA enrichment accompanied with the same trends of IBD and ND antibody responses, boosting immune system and enhance spleen task. Increasing of the IBD and ND titer can be explained by the fact that IL-2 is able to facilitate production of immunoglobulins made by β cells. Anti-sheep red blood cell antibody responses of rats fed 17 g fish oil were significantly higher than corn oil fed rats [32]. The β cell population is not affected by dietary fat in non-infected mice, but the fish oil enrichment results in the highest percentage of β cells in infected mice [33]. Furthermore, fortifying the diet with *n-3 *PUFA from fish oil (5%) significantly increased serum IgG concentration and IgM+ B cells in chicken [30]. Immunoglobulin M as the major antibody in the primary immune response reduces in circulating system during the acute lytic phase of the IBD virus [8,10,11]. The present study provides the evidences for immuno-stimulating properties of dietary n-3 PUFA in IBD infected chicken through antibody body response improvement and probably IgM-bearing B lymphocyte proliferation.

One of the interesting results of the current study is the ascending elevation of IL-2 and IFN-γ concentrations along with the *n-3 *PUFA enrichment from T2 to T4. Cytokine production plays an important role in mounting a complete and full immune response of both the innate and specific systems. These cytokines are produced by T helper lymphocytes (Th1) and activate macrophages, NK cells, and cytotoxic T lymphocytes and are the principal effectors of cell-mediated immunity. Interactions with bacteria, viruses, and fungi tend to induce Th1 activity. Because Th1 cytokines activate monocytes and macrophages, these cytokines may be regarded as pro-inflammatory. Interlukin-2 can enhance interferon production [34,35]. Interferon-γ is a cytokine that is critical for innate and adaptive immunity against viral and intracellular bacterial infections. A close relationship exists between cytokine synthesis and *n-3 *fatty acids. It is possible that eicosanoid production derived from *n-3 *PUFA might stimulate the production of IL-2 and IFN-γ [36,37]. At present, the mechanisms by which dietary *n-3 *PUFA modulate cytokine production is not clarified. However, the possible mechanism might be the decreased production of metabolites of *n-6 *PUFA, such as PGE_2 _and changes in phospholipids composition of immune cell membranes. Some studies suggested the PGE2 which is derived from arachidonic acid and *n-6 *PUFA as the possible candidate for inhibition of T cell proliferation, Th1 cell, IL-2 and IFN-γ production [38-40]. The PGE_2 _primes human naive T cells in a dose-dependent fashion for production of high levels of IL-4, IL-10, and IL-13 and very low levels of IL-2 and IFN-γ [41]. Furthermore, some researchers noted that high levels of dietary fish oil apparently have different immunomodulatory effects than lower levels [42]. It is reported that *n-3 *PUFA decreases production of PGE_2 _[43] due to a competition between *n-3 *PUFA and *n-6 *PUFA for incorporation into the cell membrane phospholipids. This finding is in contrast with the result of dietary fish oil supplementation in human [4,36] and rat [44]. However, these findings are consistent with the effects of dietary supplementation in non-human primates [45] in human [43,46] and mice [47]. A possible explanation for the contrast in results between the present study and other reported studies may referred to the intake levels EPA and DHA, source and type of oil and the concentration of dietary fat. There is another highlight in the current study results on the IL-2 and IFN-γ concentration where high level of these cytokines observed in T1 chickens that received the lowest amount of n-3 PUFA. There is no clear explanation for the phenomenon, but it may speculated that this observation is contributed to the dose-response characteristic of the immune functions to n-3 PUFA levels [41,42] or low level of linoleic acid in T1 compare to the other groups. Linoleic acid reported to inhibit the proliferation of rodent and human lymphocytes and decrease the production of IL-2 by mitogenstimulated rat and human lymphocytes [5,48,49], suggesting that it is potentially immunosuppressive.

## Conclusions

In conclusion, dietary n-3 PUFA enrichment may improve the immune response and IBD resistance, but the optimum performance does not coincide with the optimum immune response. It seems that dietary n-3 PUFA modulates the broiler chicken performance and immune response to IBD challenge in a dose-dependent manner. Thus, a moderate level of dietary n-3 PUFA enrichment may help to put together the efficiency of performance and relative immune response enhancement in broiler chickens.

## Methods

### Birds and Housing Environment

A total of three hundred day-old male broiler chicks (Cobb 500) were obtained from a local hatchery. The chicks were wing-banded, individually weighed, and housed. The birds were raised on conventional deep litter system in an open sided house with cyclic temperatures (minimum, 24°C; maximum, 34°C). The relative humidity was between 60 to 80%. The area of each pen was 2 m^2^. Feed and water were provided *ad libitium *and lighting was continuous. The chicks were vaccinated against Newcastle disease (Animal Health, Fort Dodge, Iowa, USA) on day 7 (intraocular) and on day 21(intranasal).

### Experimental Design

Experimental procedure was approved by the ACUC Animal Care and Use Committee of the University of Putra Malaysia. Commencing from day one, five replicate pens of 15 chicks each were assigned to one of the four dietary treatments, giving a total of 20 pens. There were four dietary *n-3 *PUFA ascending levels as the treatment groups (T1: 0.5; T2: 8.0; T3: 11.5; T4: 16.5) achieved using a combinations of tuna oil, sunflower oil and palm oil in feed formulation (Table 1). All diets were isocaloric and isonitrogenous. The diets (mash form) were formulated to meet or exceed requirements by the NRC [50] for broiler chickens. The diets were prepared weekly and kept at 4°C to prevent rancidity and oxidation. No antimicrobial, anticoccidial drugs or feed enzymes were included in the diets.

### Performance Parameters

All chickens were individually weighed weekly. Feed intake per group was recorded weekly and feed conversion ratios (FCR) were calculated. Mortality was recorded daily in each subgroup.

### Challenge Protocol

On day 28 of age, all birds were challenged by an oral route with a commercial live IBD vaccine (V877 strain, Malaysian Vaccines and Pharmaceuticals Sdn. Bhd). The strain was characterized as an intermediate classical strain. Each bird was inoculated with a dose of 10^4.0 ^EID_50 _IBD virus into the lumen of the crop by oral gavage [51].

### Lymphoid Organ Weight and Histopathological Examination

Prior to IBD challenge (d 28), 7 and 14 d post-challenge, eight birds from each group were chosen randomly and killed by cervical dislocation, the bursas of Fabricius and spleen were removed and their weight recorded and presented as relative to body weight [52]. The bursas of Fabricius were then fixed in 10% buffered formalin, processed for histology, and lesions were scored from 0 to 5 according to the modified criteria described by Muskett [53]. The breast muscle samples also were collected and frozen quickly in liquid nitrogen, and stored at -70°C until further analysis for fatty acids profile.

### Fatty acid Extraction and Identification

The total fatty acids were extracted from diets, oils and breast muscle samples using chloroform: methanol 2:1 (v/v) based on the Folch [54] modified by Rajion [55] with an antioxidant to prevent the oxidation during sample preparation. The experimental diets and plasma were homogenized in 40 ml chloroform: methanol (2:1 v/v) using an Ultra-Turrax T5 FU homogenizer (IKA Analysentechnik GmBH, Germany). Transmethylation of the extracted fat to fatty acid methyl esters (FAME) were carried out using KOH in methanol and 14% methanolic boron triflouride (BF_3_) (Sigma Chemical Co. St. Louis, Missouri, USA) according to methods in AOAC [56]. The methyl esters were quantified by gas chromatography (Agilent 7890N) using a 30 m × 0.25 mm ID (0.20 μm film thickness) Supelco SP-2330 capillary column (Supelco, Inc., Bellefonte, PA, USA). One microliter of FAME was injected by an auto sampler into the chromatograph, equipped with a split/splitless injector and a flame ionization detector (FID). The injector temperature was programmed at 250°C and the detector temperature was 300°C. The column temperature program initiated runs at 100°C, for 2 min, warmed to 170°C at 10°C/min, held for 2 min, warmed to 220°C at 7.5°C/min, and then held for 20 min to facilitate optimal separation. All results of fatty acid were presented as the percentage of total fatty acids. All peaks were quantified using fatty acid standards (Supelco 18919, fatty acid methyl ester mixture, USA).

### Serology

Prior to IBD challenge (d 28), 7 and 14 d post-challenge, ten birds from each group were chosen at random, and blood samples were collected from the brachial vein. Serum was separated by centrifugation (3000 g, 15 minutes) and antibody titre against ND and IBD were measured using commercially available ELISA kits (Bio-check B.V., Gouda, Holland) according to manufacturer's instructions. The absorbance of controls and samples were read at 405 nm using an ELISA reader (Bio-Tek Instruments Inc. ELX 800; Winooski, VT).

### Cytokine Measurement

The serum levels of IL-2 and IFN-γ were determined using a chicken specific ELISA kits (Cusabio Biotech, USA). The procedure was following the procedure recommended by the manufacturer.

### Statistical Analysis

A completely randomized design (CRD) with five replicates was employed for this study. Statistical analyses were conducted using one-way ANOVA general linear models. Means were separated by Duncan's multiple range tests. Antibody titers of IBD and ND were logarithmically transformed prior to analysis to achieve homogeneity of variance and were expressed as log_10_. The IL-2 and IFN-γ data were normalized using square root transformation prior to analysis. Mortality data were subjected to chi-square analysis. The results were expressed as mean ± SEM. Statistical significance was considered at P < 0.05.

## List of Abbreviations

IBD: infectious bursal disease; ND: Newcastle disease; IL: interleukin; IFN: Interferon; ELIZA: Enzyme-linked immunosorbent assay; SRBC: sheep red blood cell; PGE2: prostaglandin E2; PUFA: polyunsaturated fatty acid.

## Competing interests

The authors declare that they have no competing interests.

## Authors' contributions

EM, AFS and ME conceived and designed the study, participated in data collection and analyses and drafted the manuscript; AK, TCL, FH, MHB participated in the design of the study and drafted the manuscript. YMG participated in the data analyses and fatty acid analysis; TZ helped for the interpretation of the histopathological examination. All authors read and approved the final manuscript.
